# Urinary excretion kinetics of [^177^Lu]Lu-PSMA-617

**DOI:** 10.1007/s00259-023-06328-8

**Published:** 2023-07-08

**Authors:** Maarten de Bakker, Noa Dominicus, Antoi Meeuwis, Marcel Janssen, Mark W. Konijnenberg, James Nagarajah, Steffie M. B. Peters

**Affiliations:** 1 Department of Medical Imaging, Radboud University Medical Center, P.O. Box 9101, 6500 HB Nijmegen, The Netherlands; 2 Department of Radiology and Nuclear Medicine, Erasmus Medical Center, Rotterdam, The Netherlands

**Keywords:** PSMA, Excretion kinetics, Radiation safety, Therapy

## Abstract

**Introduction:**

For the implementation of suitable radiation safety measures in [^177^Lu]Lu-PSMA-617 therapy, additional insight into excretion kinetics is important. This study evaluates this kinetics in prostate cancer patients via direct urine measurements.

**Methods:**

Both the short-term (up to 24 h, *n* = 28 cycles) and long-term kinetics (up to 7 weeks, *n* = 35 samples) were evaluated by collection of urine samples. Samples were measured on a scintillation counter to determine excretion kinetics.

**Results:**

The mean excretion half-time during the first 20 h was 4.9 h. Kinetics was significantly different for patients with kidney function below or above eGFR 65 ml/min. Calculated skin equivalent dose in case of urinary contamination was between 50 and 145 mSv when it was caused between 0 and 8 h p.i.. Measurable amounts of ^177^Lu were found in urine samples up to 18 days p.i..

**Conclusion:**

Excretion kinetics of [^177^Lu]Lu-PSMA-617 is especially relevant during the first 24 h, when accurate radiation safety measures are important to prevent skin contamination. Measures for accurate waste management are relevant up to 18 days.

## Background

In [^177^Lu]Lu-PSMA-617 therapy for metastasized prostate cancer, both the external dose rate of patients and the risk of radioactive contamination for caregivers and relatives are mainly determined by the urinary excretion of [^177^Lu]Lu-PSMA-617. However, the knowledge about the excretion kinetics is limited and is derived from data from either external dose rate measurements [1] or urine samples of a small number of patients and at limited time points [2]. Furthermore, information on long-term excretion kinetics (> 4 days post injection) is lacking. This information is important since this could create possible contamination risks in case of rehospitalization or the need for other medical interventions. Thus, for the implementation of suitable radiation safety measures, additional insight into this kinetics is important. This study evaluates the [^177^Lu]Lu-PSMA-617 excretion kinetics in prostate cancer patients via direct urine measurements, including both hormone-sensitive (HSPC) and castrate-resistant (CRPC) patients.

## Methods

### Experimental setup short-term excretion kinetics

Patients receiving [^177^Lu]Lu-PSMA-617 therapy were asked to collect their urine in separate flasks during hospitalization (up to 24 h p.i.). Flasks were weighted and 1 ml samples (in triplets, also weighed) from each micturition were measured in a scintillation counter (248 WIZARD^2^, PerkinElmer, Groningen, The Netherlands) that was calibrated for ^177^Lu. An excretion curve for each patient was determined, and average kinetics were calculated to determine the prognosed excretion at later time points (e.g., 48 h).

A total of 30 therapy cycles of [^177^Lu]Lu-PSMA-617 were initially included for evaluation, two of which had to be excluded because urinary collection was unsuccessful. Three patients were included twice to evaluate inter-cycle variability. Exclusion criteria were kidney function (eGFR) < 50 ml/min and any severe incontinence. For further demographics, see Table 1. Excretion kinetics are compared between HSPC and CRPC patients, as well as for patients with varying kidney function, to verify a possible correlation (unpaired *t*-test). Also, total miction volume as a function of total excretion at the time of discharge was analyzed.

**Table 1:** Patient demographics

		Short-term excretion median (range)	Long-term excretion median (range)
*N*	Total	25	9
	HSPC	17	0
	CRPC	8	9
Age (years)		69 (48–77)	69 (62–76)
Cycle		1–4	1–6
Time collected p.i		18.1 (12.7–21.0) h	17 (7–41)
Number of mictions		8 (5–14)	n/a
Miction volume (ml)		1800 (500–4000)	n/a

### Risk of contamination and related skin dose

The equivalent skin dose in case of contamination during the first 48 h was calculated based on the average excretion kinetics (Online Resource 1). Administration of an activity of 7.4 GBq [^177^Lu]Lu-PSMA-617 was assumed, and contamination with a 50 µl droplet on 1 cm^2^ of skin, which was mostly removed within 5 min. A remaining activity of 5% was assumed on a 5 cm^2^ surface [3, 4].

### Experimental setup long-term excretion kinetics

To evaluate the presence of ^177^Lu in urine at later time points, CRPC patients that received [^177^Lu]Lu-PSMA-617 therapy were asked to collect a urine sample during regular hospital check-ups. A total of 35 samples were collected at 7 to 42 days p.i., originating from 9 patients and 16 different treatment cycles. Activity concentration in each sample was determined in triplets on the same scintillation counter as described above. Since no information on total miction volume was available, the data was only used to determine the time period in which measurable amounts of ^177^Lu were still present in the urine.

## Results

### Excretion kinetics

**Fig. 1:**
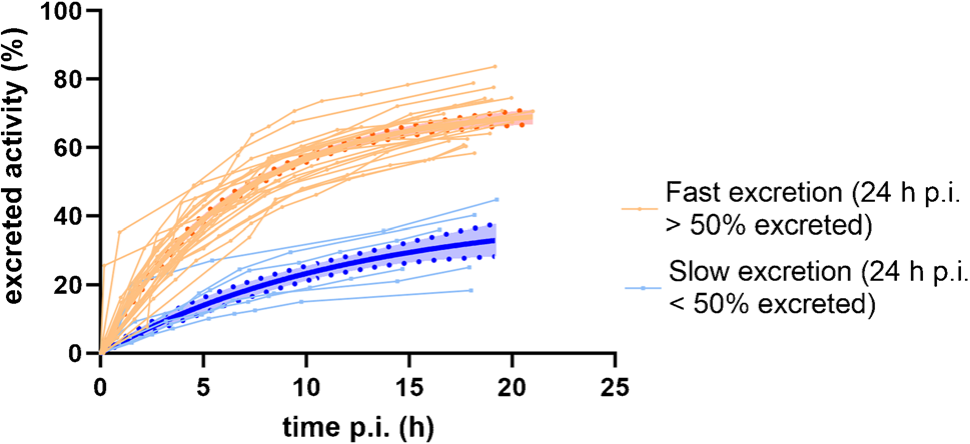
[^177^Lu]Lu-PSMA-617 excretion kinetics during the first 24 h for 25 patients, 28 therapies

Two different excretion patterns for [^177^Lu]Lu-PSMA-617 were identified (Fig. 1), showing > 50% or < 50% excretion during the first 24 h, respectively. The mean excretion half-time during the first 20 h was 4.9 h for the total group, 4.4 h for the ‘fast excretion’ group (*n* = 19), and 8.4 h for the ‘slow excretion’ group (*n* = 6) (significantly different: *p* < 0.01). The prognosed excretion at 48 h p.i. was 67 ± 18% for the total group, and 75 ± 10% and 36 ± 10% for the fast and slow groups, respectively.

**Fig. 2:**
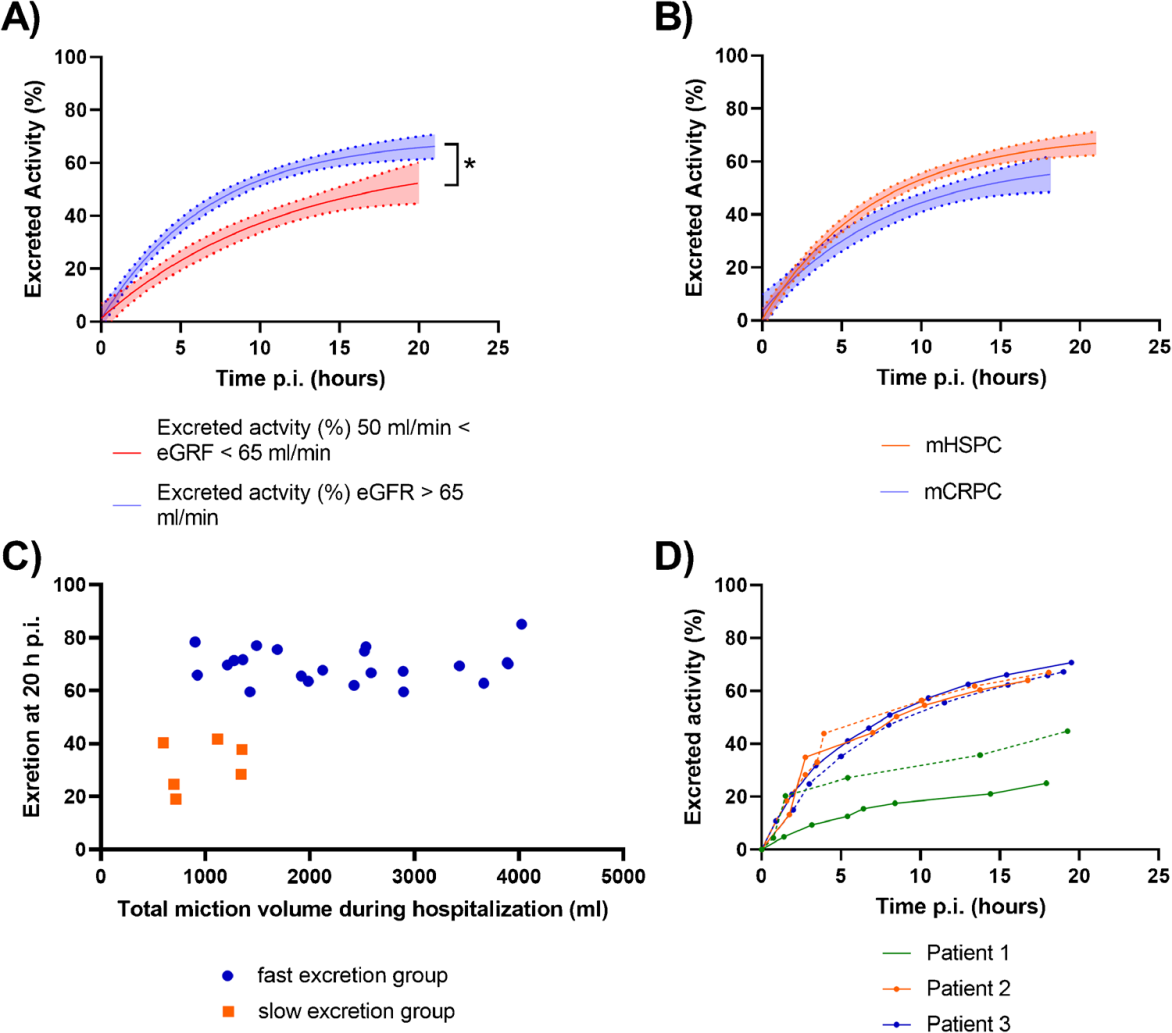
Correlation between [.^177^Lu]Lu-PSMA-617 excretion kinetics and various parameters. **A** Excretion kinetics for different kidney functions (significantly different). **B** Excretion kinetics for HSPC and CRPC patients. **C** Excretion at 20 h p.i. (%) as a function of total miction volume, for fast and slow excretion group. **D** Excretion kinetics for three patients that were followed for 2 different therapy cycles (solid line: cycles 1–2, dashed line: cycles 3–4)

Excretion for patients with a kidney function between 50 ml/min < eGRF < 65 ml/min (*n* = 7) was significantly slower than for patients with a kidney function of eGRF > 65 ml/min (*n* = 21, *p* < 0.01) (Fig. 2A). There was no significant difference in excretion between HSPC and CRPC patients (*p* = 0.07) (Fig. 2B). When comparing total miction volume during hospitalization to excretion at 20 h p.i. (%), the slow excretion group had a significantly lower miction volume (*p* < 0.01) (Fig. 2C). Three HSPC patients were included for 2 different therapy cycles (Fig. 2D). For 2 patients, excretion kinetics were almost identical between cycles. For the other patient, total excretion at 20 h p.i. was 25% and 45% for cycles 2 and 4, respectively.

**Fig. 3:**
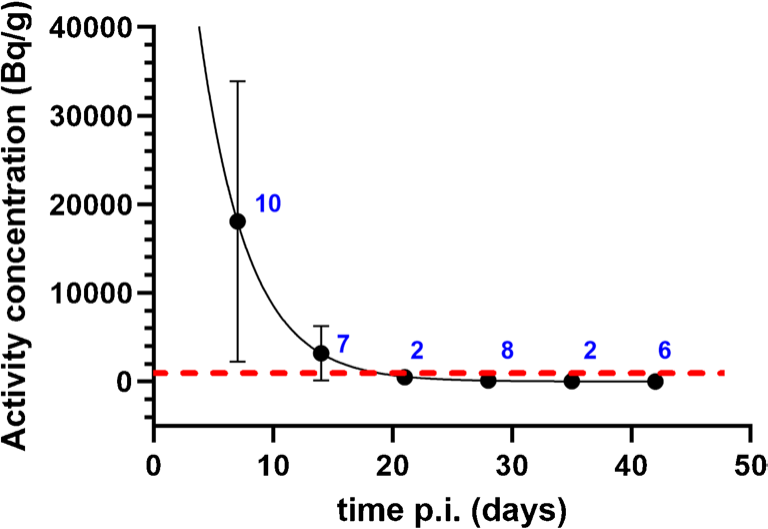
Long-term [^177^Lu]Lu-PSMA-617 excretion kinetics. The red line indicates the 1 kBq/g limit, which holds as the exemption limit in many countries. Number of samples collected per time point are indicated in blue

The amount of ^177^Lu present in urine at later time points varied largely between patients, especially at 7 days post injection (Fig. 3).

### Skin dose in case of contamination

**Fig. 4:**
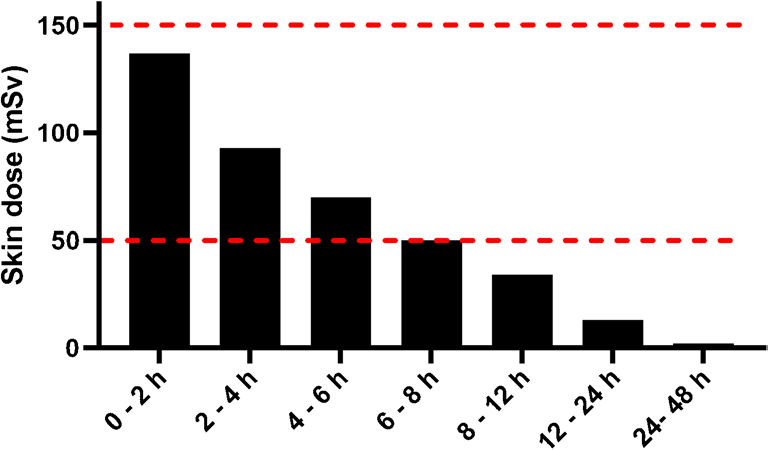
Skin dose in case of contamination with a 50 µl droplet of urine, for time intervals p.i. as indicated on x-axis. Red lines indicate both 50 and 150 mSv skin equivalent dose

Skin dose in case of contamination decreased quickly during the first 48 h (Fig. 4). When a contamination was caused between 0 and 8 h p.i., the total skin equivalent dose was between 50 and 145 mSv.

## Discussion

This study evaluated both the short- and long-term excretion kinetics of [^177^Lu]Lu-PSMA-617 therapy in prostate cancer patients by using direct urine measurements. A large variation between patients was found. For the majority of patients, excretion kinetics during the first 24 h were similar to those found in earlier studies (1, 2), with a mean half-time of 4.9 h. However, we also identified a subgroup of patients that showed significantly slower excretion kinetics. It was striking that all these patients had a small total miction volume during hospitalization, indicating that there might be an effect on excretion kinetics if patients consume less fluid. While some patients with regular excretion kinetics had a similarly small miction volume, we suggest that sufficient fluid intake after therapy is important as this will likely lead to increased miction volume and could potentially stimulate [^177^Lu]Lu-PSMA-617 excretion.

There was a significant difference in excretion kinetics between patients with different kidney functions, indicating limited kidney function might hamper fast excretion of PSMA. Of course, in this study no patients were included with a clinically relevant decreased kidney function of eGRF < 50 ml/min, which might have an even stronger effect on the excretion kinetics. No significant difference was found between HSPC and CRPC patients. What would be of interest, however, is to compare patients of different tumor load to evaluate the effect of a possible sink effect. This was not possible in this study since up-to-date information on total tumor volume was not available for most patients.

Variation in excretion kinetics between therapy cycles within the same patient was very low for 2 out of 3 patients, indicating that there might be a limited effect of earlier therapies on excretion kinetics. For 1 patient, however, excretion kinetics differed between cycles, while total miction volume, number of mictions, and kidney function (eGFR) were very comparable between the cycles. Since this was only found in one patient, more data would be needed to evaluate if this effect would be found more regularly.

The long-term excretion kinetics up to 41 days showed large variation between patients, especially at 7 days p.i., which can at least partially be explained by differences in bladder filling at the time of sampling. However, at 7 days p.i., all patients had ^177^Lu present in the urine at levels that are relevant to consider for radiation safety measures. This holds for the prevention of skin contamination of personnel and caregivers, but also for accurate waste management. Since many countries work with an exemption limit of 1 kBq/ml for ^177^Lu, we suggest the implementation of relevant radiation safety measures up to 18 days p.i., especially when rehospitalization is indicated.

At earlier time points, skin contamination with a droplet of urine can lead to a significant skin equivalent dose, especially during the first 8 h after therapy. Since many patients in this population suffer from incontinence or need a catheter, personnel might need to perform tasks that hold a significant risk of skin contamination. Therefore, it is of utmost importance to implement radiation safety measures to protect personnel by minimizing bare skin during these tasks, for example by wearing long-sleeved aprons and appropriate gloves.

## Conclusion

Excretion kinetics of [^177^Lu]Lu-PSMA-617 in prostate cancer patients is relevant, especially during the first 24 h after therapy (mean half-life 4.9 h), during which time possible skin contamination can lead to a significant skin equivalent dose and appropriate radiation safety measures are important to protect personnel. Some patients show slower excretion kinetics, which might be related to kidney function, and also partially be related to fluid intake and corresponding miction volume after therapy. Since ^177^Lu was also found present at much later time points after therapy, appropriate radiation safety measures related to waste management should be taken up to 18 days post injection.

## Data Availability

The datasets generated during and/or analyzed during the current study are available from the corresponding author on reasonable request.
